# The Expression of MMP-2 Following Immobilization and High-Intensity Running in Plantaris Muscle Fiber in Rats

**DOI:** 10.1100/tsw.2006.107

**Published:** 2006-05-05

**Authors:** Eli Carmeli, Tal Gal Haimovitch

**Affiliations:** Department of Physical Therapy, Sackler Faculty of Medicine, The Stanley Steyer School of Health Professions, Tel Aviv University, Ramat Aviv, Israel

**Keywords:** skeletal muscle, MMP-2, immobilization, running, physical activity, inactivity, muscle, enzyme, Israel

## Abstract

The effect of 2-week, high-intensity running and a 2-week immobilization on muscle fiber type composition of the plantaris muscle from 18 female, 6-month-old Wistar rats (running, n = 6; immobilization, n = 6; sedentary control, n = 6) was bio- and histochemically investigated. The high-intensity treadmill running began with 20 min (32 m/min, 0% gradient, 75% VO_2_ max), up to 50 min/day. Right hind limbs were immobilized by an external fixation procedure for 13 days. Muscle mass of the plantaris muscle in the immobilized groups was reduced by 16% in comparison with the sedentary control group. High-intensity running and immobilization increased both mRNA and protein levels of matrix metalloproteinase type 2 (MMP-2) in plantaris. Running and immobilization decreased the percentages of transverse sectional area of fast-twitch glycolytic (FG) type IIb fibers, running increased relative cross-sectional area of fast-twitch oxidative glycolytic (FOG) type IIa muscle fibers, whereas immobilization increased relative cross-sectional area of slow-twitch oxidative (SO) muscle fibers (type I). Our results suggest that both high-intensity running and immobilization are enough to induce overwhelming changes in plantaris.

### INTRODUCTION

The skeletal muscle is an adaptive tissue that rapidly undergoes changes after both overuse (i.e., intense exercise, high-speed running) and disuse, as well as under pathological conditions. Overuse and disuse might both result in significant damage to the skeletal muscle morphologically, biochemically, and functionally[1]. Matrix metalloproteinases (MMPs) are a family of at least 24 proteolytic enzymes that belong to a large group of zinc proteolytic enzymes. MMPs are crucially involved in the turnover of extracellular matrix (ECM) components[2]. MMP-2 was shown to play a key role in maintaining structure and activity of basement membrane (BM) components during muscle pathologies such as inflammatory myopathies[3,4]. While the role of MMP-2 in development, growth, and repair of skeletal muscles has been vastly investigated, its potential role during immobilization and recovery of skeletal muscles has been addressed only in a few studies[5,6].

The plantaris muscle is composed of mixed fibers. The mixed-fiber distribution makes it easier to observe specific histochemical changes in enzyme activity than in muscles of primarily one fiber type such as soleus, which contains predominantly type I fibers. Therefore, the plantaris muscle represents a mixed-fiber skeletal muscle model to ascertain overuse- and disuse-associated alteration in fiber distribution[7,8].

The purpose of this study was to test how high-intensity running and immobilization influence the expression of MMP-2 and fiber type composition of plantaris muscle in young and healthy rats.

### MATERIAL AND METHODS

Pathogen-free, female Wistar rats (6 months old, body weight range 260–320 g) were maintained under constant conditions of room temperature (22°C) and humidity (40%) with a 12/12-h night-day cycle and fed standard rat chow and water *ad libitum*. All animals were maintained according to the principles of laboratory animal care formulated by Tel Aviv University (Israel) and the experimental procedures received approval from the Ethics Committee for Experimental Procedures of Tel-Aviv University.

#### Experimental Procedure

Rats were randomly assigned to one of three groups: (1) high-intensity exercise (n = 6), (2) immobilized right hind limb (n = 6), or (3) nonexercising control (n = 6). Animals in the exercise group were acclimatized to treadmill running during a 3-day habituation period at a low intensity of exercise (10 min/day at 15 m/min, 0% grade, ~40% $VO_2$ max). This habituation period was followed by 2 weeks of treadmill running (5 consecutive days/week) according to the following protocols. High-intensity exercised animals began with 20 min of running (32 m/min, 0% grade, ~ 70–75% $VO_2$ max) with 10 min added each day until 50 min were achieved. All animals were exercised at the same time each day. The running was done in the active (dark) phase of the day cycle. These exercise intensities were chosen to provide a wide difference in exercise intensities to resolve the impact of exercise intensity on the expression of MMPs in skeletal muscle. Finally, the $VO_2$ max of the animals and speeds chosen to present each relative workload were selected based on previous work from our laboratory[9].

#### External Fixation

Rats were anesthetized by 60 mg/kg Ketamine HCl intramuscular and 40 mg/kg Nembutal intraperitoneal (i.p.) injection. Antibiotic, 70 mg/kg Cefamizine, injected with Nembutal. The external fixation (EF) model has been described by Reznick et al.[5] and Bar Shai et al.[10]. Briefly, rigid immobilization was achieved by inserting two 0.8-mm diameter Kirschner wires through the lateral plane of the femur and tibia, which were then connected by two threaded brass rods to make a rigid frame. The rods were 4.8 mm in diameter and 33 mm in length and had a 13-mm slot cut longitudinally from both ends to contain the wires. The right knee joint was immobilized in 45º flexion. The overall weight of the device was 12 g. The contralateral intact hind limb muscles were used as internal control.

Rats were sacrificed 2 weeks after the last running session and the EF. An intra peritoneum injection of penthobarbitol sodium (200mg/kg) was performed for reaching a general anesthesia (i.e., resulting in absence of lid and corneal reflex), followed by the surgical procedure of carefully separating and removing the right and left Plantaris muscles. Muscles weighed and frozen in isopentane were chilled by liquid nitrogen (–192ºC)

### RT-PCR

Total RNA was isolated from 30-mg muscle tissue using SV total RNA isolation kit (cat. 20-400-100, Biological Industries, Beit Haemek, Israel). RNA was used as a template for RT-PCR reaction (Access Quick™ RT-PCR system, Promega A1702) using MMP-2 primers: sense (Forward) CCATCAAACGGGTATCCATC and antisense (Backward) GTCGGACCTCTCAGGGTTCT; and alpha-tubulin primers (as a reference): sense ATCACAGGCAAGGAAGATGC and antisense ATTGACATCTTTGGGGACCA (Sigma). The RT-PCR products were separated on 1.2% agaros gel. Control blots were performed using only secondary antibody.

### SDS-PAGE and Western Blot Analysis

Muscle tissue (100 mg) was homogenized (20-sec homogenization and 10-sec pause × 3 times) in cold buffer containing 42 m*M* Trizma base, 0.3 *M* KCl, 2.5 m*M* MgCl, 0.1% Triton x-100, and protease inhibitor cocktail (P-8340, Sigma), and centrifuged (10,000 × *g* for 10 min at 4°C). The supernatants were collected and total protein concentration was measured using Bradford reagent (cat. 500-0006, Bio-Rad, Hercules, CA). Equal amounts of supernatants were suspended in protein sample buffer containing 5% beta-mercaptoethanol, vortexed, boiled, and centrifuged. The supernatants were treated with 10% SDS-PAGE. Proteins from polyacrylamide gels were transferred onto nitrocellulose membranes. Blots were blocked with 2.5% skim milk (cat.170-6404, Bio-Rad) in PBST (PBS containing 0.05% Tween 20) for 1 h, reacted with MMP-2 specific goat polyclonal antibody (sc-6838, Santa Cruz Biotechnology, CA) and alpha-tubulin specific mouse monoclonal IgG2a antibody (sc-5286, Santa Cruz Biotechnology, CA) for 1 h, washed three times with 2.5% skim milk in PBST for 30 min (3 × 10 min), reacted with bovine antigoat IgG-HRP (sc-2350, Santa Cruz Biotechnology, CA) or donkey antimouse IgG-HRP (sc-2314, Santa Cruz Biotechnology, CA), respectively, for 30 min, and washed once with 2.5% skim milk in PBST for 10 min and three times with PBST for 9 min (3 × 3 min). The membranes were developed using Super Signal West Pico chemiluminescent substrate (cat. 34080, Pierce Chemical Co., Santiago, Chile) followed by exposure to X-ray films (Fuji). Quantification of MMP-2 was performed using the Scion Image Version 4.0.2 beta, Scion Cooperation.

### Histochemical Procedure

Sequential slices of plantaris muscles were cut on a freezing microtome at a thickness of 8–10 µm and prepared for histochemical fiber type analysis. The enzyme stains were succinic dehydrogenase (SDH) and alkaline (pH 9.4) myosine adenosine triphosphatase (mATPase). These stains are appropriate for identifying and allow the distinction of the type I, IIa, and IIb muscle fibers.

Approximately 90 to 100 muscle fibers were counted from a Polaroid photograph taken of the muscle section–prepared slide on a standard multistage Zeiss universal microscope, CUE-2 image analysis system (Sony Corp, Santa Clara, CA) at varying magnifications. This means of analysis allowed for ease of visibility, because the photograph can be more magnified than microscopic visualization of the tissue under a high-powered lens. Independent observers skilled in neuromuscular anatomy verified the determination of the distribution of type I, IIa, and IIb muscle fibers. In addition, to measure fiber sizes in cross-sectional areas, we performed a histomorphometric analysis. Briefly, this computer system is composed of a light microscope (Zeiss M14), a video panasonic WV-CD 50, a line printer (Kennedy model 9110), and PC-IBM-AT system. An array of picture elements (640 × 480 pixels) were quantified to 256 grey levels that were then converted to optical density ranging from 0.002–2.0. The fibers were typed as darkly or lightly staining for myosin ATPase based on their optical density. Fiber cross-sectional area was determined and the reaction products from the SDH histochemical reaction were quantified. Interrelationships between fiber size and enzyme activities were determined from these samples.

### Statistical Analysis

A one-way analysis of variance was used to determine if group differences existed in skeletal muscle MMP-2 levels. Significance was established at $p < 0.05$.

## RESULTS

The effects of 2 weeks running and 2 weeks of immobilization on body weights and plantaris muscle weights are reported in Table 1. No differences existed in animal body weights between the two experimental groups at the beginning of the experiments, but 2 weeks of immobilization resulted in a decrease in plantaris muscle weight (reduced in 16%, $p < 0.05$). Records of both water and caloric intake of the animals during the experimental period revealed no group differences, suggesting that muscle weight differences were not due to dehydration or malnutrition.

**TABLE 1**
**Body Weights, Muscle Weights, Western Blot, and RT-PCR for MMP-2 in Plantaris Muscle Following 2 Weeks of Running or 2 Weeks of Immobilization**

| Method | Control | Running | % | *p | Immobilization | % | *p |
|---|---|---|---|---|---|---|---|
| Body weight (g) | 279 ± 25 | 272 ± 28 | –2.5 | NS | 270 ± 30 | –3.2 | NS |
| Muscle weight (mg) | 313 ± 31 | 316 ± 28 | –2 | NS | 271 ± 25 | –16 | 0.05 |
| Western Blot (μg/mg muscle weight) | 47 ± 4.2 | 82 ± 7.9 | +42 | 0.05 | 52 ± 4.9 | +10 | NS |
| RT-PCR (ng/ml) | 5.1 ± 0.5 | 9.6 ± 0.8 | +46 | 0.05 | 5.7 ± 0.5 | +10 | NS |

*Significantly different t $p < 0.05$ in comparison to control.

### Biochemical Analysis

To address the question of whether 2 weeks of high-intensity running or 2 weeks of immobilization resulted in significant changes in muscle levels of MMP-2, we measured muscle levels of both mRNA and protein for this important protein. MMP-2 mRNA levels significantly ($p < 0.05$) increased in plantaris muscles (+46%) (see Table 1) following speed running, whereas following 2 weeks of immobilization, the level of mRNA of MMP-2 was slightly (not significant) increased (+10%). Consistent with the mRNA results, MMP-2 protein levels were also elevated in the plantaris following the running and immobilization period, and a total of 42% ($p < 0.05$) and 10% increased (p = not significant), respectively (see Table 1).

### Enzyme Histochemistry of the Plantaris Muscle

The plantaris is composed of mixed-fiber composition. The composite distribution of cross-sectional areas for type IIa (known as fast-twitch oxidative, glycolytic, FOG), type IIb (from a motor units point of view, they were classified as fast-twitch glycolytic, FG), and type I (known as slow-twitch oxidative, SO)

plantaris muscle fibers of running, immobilization, and sedentary control animals are demonstrated in Fig. 1. The figure depicts composite sedentary control/contralateral leg of the immobilized group, high-intensity running, and immobilization-associate effects on type I, IIa, and IIb muscle fibers in plantaris as determined by the stain for SDH. The mATPase staining fully supports the SDH stain finding, therefore, mATPase stain is not presented. Running and immobilization significantly ($p < 0.05$) decreased the percentages of the transverse sectional area of FG type IIb fibers. The running intervention resulted in a significant increase ($p < 0.05$) in the percent of type IIa fibers (FOG), whereas immobilization results in slight increase ($p < 0.05$) in the percent of type I fibers (SO). The percent of type I, IIa, and IIb fibers in the contralateral leg in the immobilized group was equivalent to sedentary control percentages.

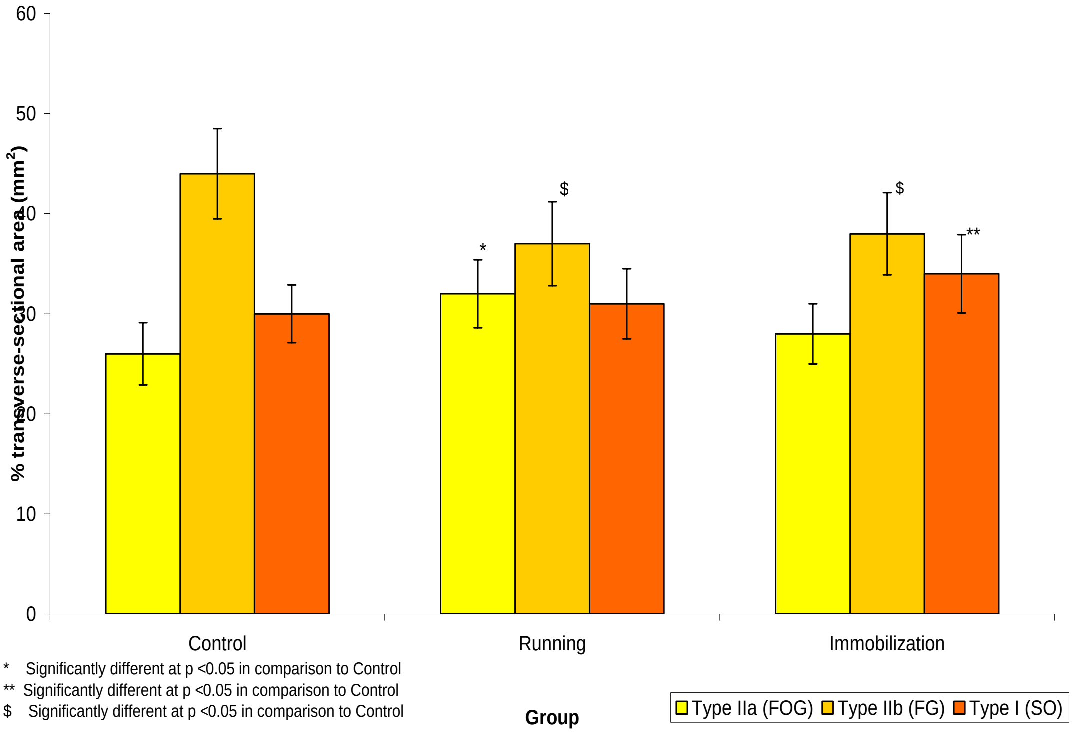

**FIGURE 1.** The effects of 2 weeks immobilization and 2 weeks of high-intensity running on type I, IIa, and IIb muscle fibers in plantaris as determined by the stain for SDH.

### DISCUSSION

The primary finding of the present study was that the plantaris muscle of female rats, particularly the type IIb fibers, undergo changes in response to high-speed running or immobilization. Although forced high-intensity treadmill running or immobilization are useful models for studying skeletal muscle plasticity in rats, these regimens undoubtedly include stress to the animals, and thus it is not obvious whether the observed physiological changes are due to stress, exercise, or a combination. However, (1) animals in the running group were acclimatized to treadmill running during a 3-day habituation period at a low intensity of exercise, (2) the rats ran during their normal active hours, and (3) running always occurred in the same cage in which the rats exercised, therefore the running condition and environment were least stressful. Thus, these conditions provide a more controlled and physiological environment, and thereby facilitate the interpretation of the observed adaptations relative to the adaptation-inducing event, i.e., high-speed running.

To our knowledge, these are the first experiments to investigate the impact of high-intensity running (i.e., overuse) vs. immobilization (i.e., disuse) on the fiber-type composition and the expression of MMP-2 of the plantaris muscle. These experiments were designed to test the hypothesis that both contradictory conditions induced expression of MMP-2 and changes in muscle fiber types. Our results support this hypothesis and, indeed, data analysis revealed that 2 weeks of high-intensity running or 2 weeks of immobilization increased the expression of MMP-2 (but insignificantly following immobilization) in plantaris muscles containing mixed-fiber types. The intervention of running resulted in a significant increase in the percentages of plantaris FOG, type IIa fibers, and the intervention of immobilization resulted in a small, but insignificant, increase of plantaris SO, type I fibers.

The implementation of 2 weeks of running program or 2 weeks of hind limb immobilization resulted in the expected "no change" in body weight. Declines in the masses of the plantaris muscles suggest that short-term limb immobilization, an extreme form of disuse, results in muscle atrophy and a decreased capacity to oxidize both carbohydrate and lipid substrate in the affected limb[10]. On the other hand, a power-training activity used in this study did not elicit muscle fiber hypertrophy, and the mean absolute plantaris muscle weight was comparable to control group.

### Immobilization and Running-Induced MMP Expression in Skeletal Muscles

It is likely that the adaptation responses of muscles to overuse or disuse must affect the overall muscle metabolism and cause some changes in ECM composition. Although the expression of proteolytic enzymes is known to be associated with various myopathies and inflammatory conditions[11], their involvement in skeletal muscle remodeling in response to varying conditions of use/nonuse has received limited investigative attention[12]. Under normal physiological conditions, skeletal muscle MMP-2 levels are constitutively expressed, but relatively low in the ECM; thus, the expression of MMP-2 due to muscle overload or inactivity is possibly regulated by cytokines and growth factors such as capillary growth factor[13].

The results of the present study clearly demonstrate that high-intensity running in particular and immobilization in some degree lead to structural damage involving ECM, indicated by acceleration activities of the active form of MMP-2 and increased capacity for ECM degradation. In regard to MMP-2 activity, we recently reported that MMP-2 might be inhibited by tissue inhibitors of metalloproteinases 1 and 2 (TIMP-1, -2), which are secreted by the myofibers. The catalytic activity of MMP-2 is regulated directly by the level by TIMP-1 and 2. The TIMPs derived from myofibers are secreted into the ECM and bind to the zymogen form of MMP-2; this binding regulates the formation and levels of mature MMP-2[14]. Importantly, we recently confirmed that activity levels of TIMPs changed in parallel with activity changes demonstrated for MMP-2[15,16].

The intervention of high-speed running and immobilization resulted in a significant increase in the percentage of plantaris FOG type IIa and FO type I muscle fibers, respectively, when compared to the age-matched sedentary control animals.

The possible explanation regarding the MMP-2 expression in plantaris muscles lies behind the composition of fiber types. It is noteworthy that even though the plantaris is composed of a mixed-fiber composition, type IIB fibers are the larger and more frequent than type IIa or type I fibers and they appear to be associated with larger amounts of extracellular collagen. It is possible that fast-twitch, type IIb fibers require more MMP-2 to maintain their structural integrity than type IIa or I fibers. Moreover, fast-twitch fibers demonstrate more rapid metabolic adaptation than slow fibers and adapt faster to endurance exercise training[16]. It is also possible that, as a result of intensive exercise or immobilization, the fast fibers may undergo a transition to slow fiber types (i.e., type IIa and type I fibers)[17] with corresponding changes in the composition of the ECM[18].

### Fiber Type Composition Following Immobilization and Running

In the current study, we demonstrated that different muscle fiber types show differing response patterns to running and immobilization. We found that type IIb muscle fibers were more susceptible to fast-speed running/overuse and shifted to type IIa, whereas under immobilization/disuse, the type IIb fibers mainly shifted to a type I fibers. The results indicate that the responsiveness to overloading or inactivity differed among the fiber types, i.e., FG > FOG > SO. Hind limb immobilization resulted in a progressive decline in the FG type IIb of the plantaris muscle, and the percentage of SO type I fibers in particular, and FOG type IIa muscle fibers increased.

Our finding that high-intensity exercise promoted increased percentages of FOG (type IIa) in plantaris muscles, which are characterized with mixed-fiber composition, is supported by the findings reported by Seene et al.[19] who found a composition shift of myosin heavy chain (MHC) isoform in overtraining running program from FG (type IIb fibers) to FOG (type IIa fibers) in plantaris muscles of Wistar rats. However, contradictory findings were reported by Koskinen et al.[20]. A definitive explanation for these divergent findings is not available. Nonetheless, it seems likely that differences in the exercise protocol between the current study and the work of Koskinen et al.[20] is likely to play a role in the differing results. Indeed, Koskinen et al. employed a 1-day exercise protocol of downhill running, which produced an eccentric contraction that resulted in significant levels of acute skeletal muscle injury, whereas the protocol used in the current study was chronic (i.e., 2 weeks) treadmill running that was unlikely to promote continued levels of muscle injury. However, Koskinen et al. reported that acute downhill running has been shown to activate MMP-2.

Chronic exercise has been well documented to improve the oxidative capacity of rat skeletal muscle[21,22,23]. The exercise protocol utilized in this study was sufficient to improve the oxidative capacity of the plantaris muscle. Sullivan et al.[24] and Demirel et al.[25] have shown that endurance training in rats causes a shift in MHC isoforms from a faster to a slower MHC in plantaris muscle. From previous reports, it has been shown that fast-speed running leads to muscle tissue damage[26] followed by functional decline[27]. In plantaris muscles, net tissue degradation was observed when related to soluble protein concentration, suggesting a higher rate of protein degradation in (type II) fast-twitch muscle fibers than in (type I) slow-twitch muscle fibers.

When the mean of the fibers were related by cross-sectional area parameter, a significant increase of the area occupied by type IIa was observed following high-intensity area, and type I was observed following immobilization contrasted the decrease of area occupied by type IIb. From this fibrilar composition, it can be assumed that there was a relative decrease of glycolytic capacity or relative increase of muscle oxidative capacity. It is clear that the implementation of exercise as a lifestyle modification and avoiding deconditioning should result in better maintenance of muscle metabolism.

### Clinical Relevance

The quality of human life depends on physical activity. As we age, we are less active and little involved with physical exercise. The question of "how much exercise is enough and how much is too much" needs to be addressed. Recent interest has focused on the possible effects of running as a therapeutic option in the treatment of deconditioning. MMPs are crucially involved in the turnover of ECM due to exercise, muscle diseases, and aging. MMP research, therefore, holds great therapeutic potential and is relevant not only to basic science researchers, but also clinicians need to consider therapy in the future and to their patients.

Nonetheless, the effects of exercise on skeletal muscle tissues are dose dependent. Clinical side effects of "too much exercise" include fatigue, muscle cramping, and reversible inflammation. Our data showed that immobility and hypermobility at clinically relevant doses resulted in a profound muscle damage. We showed a decrease in the percentages of transverse sectional area, yet the whole plantaris muscle as one unit underwent different changes in different areas. Elucidating the regulation of these molecules during running and immobilization in animals may lead to a better understanding of their role in physiological and pathological processes in muscle tissue in humans.

We conclude that high-intensity running and 2 weeks of immobilization are required to promote the expression of MMP-2 in the plantaris muscle. Moreover, our results indicated that 2 weeks of high-speed running or 2 weeks of immobilization promote muscle fiber changes and that changes involve degradation and synthesis of ECM. These findings form the foundation for additional research to determine the functional significance of changes of muscle fiber composition and the expression of MMP in skeletal muscles in response to fast-speed running or immobilization.

### ACKNOWLEDGMENTS

We would like to thank Anne and Eli Shapira Charitable Foundation from Portland, Oregon, for supporting this work.

### BIOSKETCHES

**Eli Carmeli, BPT, PhD**, is currently a Senior Lecturer of Gerontology and Anatomy and the Chairman of the Department of Physical Therapy, Stanley Steyer School of Health Professions, Sackler Faculty of Medicine, Tel Aviv University. He is also the Editor-in-Chief of the Israeli Journal of the Physiotherapy Society. Eli received his BPT degree from Sackler Faculty of Medicine, Tel Aviv University and his PhD degree from the Rappaport Faculty of Medicine at the Technion in Haifa. Postdoctoral research was conducted at the University of Florida in Gainesville. His research interests are investigating the aging process both on the cellular and clinical level. E-mail: elie@post.tau.ac.il Website: http://www2.tau.ac.il/Person/medicine/HealthSchool/researcher.asp?id=agfhfiffl

**Tal Gal Haimovitch**, **BSc,** Sackler Faculty of Medicine, Tel Aviv University, Israel. E-mail: talhaimo@post.tau.ac.il
